# Generation of induced pluripotent stem cells from an individual with early onset and severe hypertrophic cardiomyopathy linked to *MYBPC3*: c.772G > A mutation

**DOI:** 10.1007/s13577-024-01073-y

**Published:** 2024-05-18

**Authors:** Marta Ribeiro, Joanna Jager, Marta Furtado, Teresa Carvalho, Joaquim M. S. Cabral, Dulce Brito, Maria Carmo-Fonseca, Sandra Martins, Simão Teixeira da Rocha

**Affiliations:** 1 iBB - Institute for Bioengineering and Biosciences and Department of Bioengineering, Instituto Superior Técnico, Universidade de Lisboa, Lisbon, Portugal; 2 Associate Laboratory i4HB Institute for Health and Bioeconomy, Instituto Superior Técnico, Universidade de Lisboa, Lisbon, Portugal; 3 Centre for Heart Muscle Disease, Institute of Cardiovascular Science, University College London, London, UK; 4 Faculdade de Medicina, Instituto de Medicina Molecular João Lobo Antunes, Universidade de Lisboa, Lisbon, Portugal; 5 Heart and Vessels Department, Cardiology Division, Centro Hospitalar Universitário de Lisboa Norte, Lisbon, Portugal; 6 Centro Cardiovascular da Universidade de Lisboa (CCUL@RISE), Faculdade de Medicina da Universidade de Lisboa, Lisbon, Portugal

**Keywords:** hiPSCs, *MYBPC3*, Hypertrophic cardiomyopathy, Disease modeling

## Abstract

Hypertrophic cardiomyopathy (HCM) is frequently caused by mutations in the *MYPBC3* gene, which encodes the cardiac myosin-binding protein C (cMyBP-C). Most pathogenic variants in *MYPBC3* are either nonsense mutations or result in frameshifts, suggesting that the primary disease mechanism involves reduced functional cMyBP-C protein levels within sarcomeres. However, a subset of *MYPBC3* variants are missense mutations, and the molecular mechanisms underlying their pathogenicity remain elusive. Upon in vitro differentiation into cardiomyocytes, induced pluripotent stem cells (iPSCs) derived from HCM patients represent a valuable resource for disease modeling. In this study, we generated two iPSC lines from peripheral blood mononuclear cells (PBMCs) of a female with early onset and severe HCM linked to the *MYBPC3*: c.772G > A variant. Although this variant was initially classified as a missense mutation, recent studies indicate that it interferes with splicing and results in a frameshift. The generated iPSC lines exhibit a normal karyotype and display hallmark characteristics of pluripotency, including the ability to undergo trilineage differentiation. These novel iPSCs expand the existing repertoire of *MYPBC3*-mutated cell lines, broadening the spectrum of resources for exploring how diverse mutations induce HCM. They additionally offer a platform to study potential secondary genetic elements contributing to the pronounced disease severity observed in this individual.

**Supplementary Information:**

The online version contains supplementary material available at 10.1007/s13577-024-01073-y.

## Introduction

iPSCs have revolutionized the field of disease modeling by offering a versatile platform for studying a wide range of disorders in a controlled laboratory setting. These cells are generated by reprogramming adult somatic cells, such as dermal fibroblasts or PBMCs, into a pluripotent state, mimicking the properties of embryonic stem cells. Once reprogrammed, iPSCs can be differentiated into various cell types, including neurons, cardiomyocytes, hepatocytes, among others [1]. One of the main advantages of iPSC-based disease modeling is its ability to recapitulate the genetic and phenotypic characteristics of individual patients. By deriving iPSCs from patients with known genetic mutations or disease phenotypes, researchers can create personalized disease models that closely resemble the patient's condition. This approach allows for the investigation of disease mechanisms, the identification of novel therapeutic targets, and the development of personalized treatments [2]. HCM is the most prevalent genetic cardiovascular disorder, affecting up to 1 in 200 individuals [3, 4]. Clinical diagnosis primarily relies on a left ventricular wall thickness of  ≥ 15 mm in the absence of abnormal loading conditions [5]. Patient symptoms can be relentlessly progressive culminating in heart failure and sudden cardiac death, underscoring the importance of developing therapeutic strategies to slow, halt or reverse disease progression [6]. Typically, HCM is inherited in an autosomal dominant manner with incomplete penetrance and high phenotypic variability, even within the same family [7]. The majority of HCM cases are caused by single allelic mutations in genes that encode cardiac sarcomere-associated proteins, and *MYH7* and *MYBPC3* stand out as the most commonly mutated genes [8]. Variants within the *MYH7* gene primarily consist of missense mutations, resulting in the production of an abnormal, activated protein. This protein integrates into the sarcomere, functioning as a deleterious poison peptide [9]. In contrast, most *MYBPC3* variants consist of nonsense, frameshift or splice site mutations that likely result in decreased levels of functional cMyBP-C protein within the sarcomeres [10–12]. However, a subset of *MYPBC3* variants are missense mutations, and the molecular mechanisms underlying their pathogenicity remains controversial. The *MYBPC3*: c.772G > A is a founder mutation originating from Tuscany, Italy, with a marked propensity to systolic dysfunction and disease progression [13, 14]. The transcript change is predicted to result in a missense substitution at position 258 (a lysine residue in place of glutamic acid, p.Glu258Lys). However, recent studies in mouse models [15, 16] and human tissues [17, 18] indicate that this variant alters the last nucleotide of exon 6 resulting in exon skipping that ultimately leads to a frameshift [19–22]. While numerous patients carrying the *MYBPC3*: c.772G > A mutation have either reported no symptoms or only mild limitations before the age of 45 [18], in this study, we focus on an individual who exhibited an early onset and a severe progression of the disease. In pursuit of unraveling the factors contributing to this atypical presentation, we generated two iPSC lines derived from the patient’s PBMCs.

## Materials and methods

### Patient recruitment and ethics committee

A blood sample was collected from the F97 patient, after obtaining written informed consent, following the ethical guidelines outlined in European and National regulations (law 12/2005) of the Lisbon Academic Medical Center Biobank (Biobanco-iMM) and receiving approval from the Ethics Committee of Lisbon Academic Medical Center (Ref. no 468/20, February 23, 2020).

### Generation and maintenance of iPSC lines

PBMCs were isolated from the whole blood using Ficoll–Paque and preserved in 20% DMSO (Merck, #D8418) in foetal bovine serum (FBS) (Life Technologies, #10500), following the standardized procedure of Biobanco-iMM. Before the reprogramming phase, thawed PBMCs were cultured for 4 days in StemPro™-34 medium (Gibco, 10639011) supplemented with specific cytokines (IL-3, IL-6, FLT-3, and SCF - Prepotech/Tebu-bio, 200.03, 200.06, 300-19, 300-07). Subsequently, 5 × 10^6^ cells were transduced using the CytoTune™-iPS 2.0 Sendai Reprogramming Kit (Thermo Fisher Scientific, A16517) with the following Multiplicity of infection (MOI) for each virus: *KOS* MOI = 5, *hc-Myc* MOI = 5 and *hKlf4* MOI = 3. On the third day post-transduction, cells were reseeded into Matrigel^®^-coated 6-well culture plates and transitioned to mTeSR™Plus Medium (StemCell Technologies, 100-0276) in the following days. Colonies exhibiting characteristic stem cell morphology were individually picked and transferred to new Matrigel^®^-coated dishes in mTeSR™Plus Medium. Continuous passaging was performed until the absence of RNA genome of the Sendai Viruses (*SeV)* was confirmed by qRT-PCR (passage 14 for clone 1F97 and passage 11 for clone 5F97). iPSCs were maintained at 37 °C in a humidified 5% CO_2_, 20% O_2_ incubator and passaged 1:3–1:6 every 3–4 days when colonies covered approximately 80% of the culture dish surface area, utilizing 0.5 mM EDTA dissociation buffer (Thermo Fisher Scientific, 15575020). Further characterization analyses of the two iPSC clones were performed between passages 11 and 20, after *SeV* clearance.

### Targeted sequencing

Genomic DNA from iPSCs was extracted using the conventional phenol:chloroform:isoamyl alcohol method. Following extraction, the DNA underwent PCR amplification with primers for the region of interest of the *MYBPC3* gene (Table S1) and subjected to Sanger sequencing, by StabVida Lda (Lisbon, Portugal).

### G-banding karyotyping

For G-banding karyotyping, iPSCs in an exponential growth phase were arrested in metaphase by exposure to colcemid (10 μg/ml; Thermo Fisher Scientific, 15212012) for 5 h at 37 °C. Subsequently, cells were harvested using Accutase (STEMCELL Technologies, 07922), treated with a hypotonic potassium chloride solution for 30 min at 37 °C, and fixed with a 1:3 (v/v) acetic acid:methanol solution. Karyotype analysis was completed by Genomed SA (Lisbon, Portugal).

### Trilineage differentiation assays

The trilineage differentiation potential of iPSCs into endoderm, mesoderm, and ectoderm was evaluated using the STEMdiff Trilineage Differentiation Kit (STEMCELL Technologies, 05230), according to the manufacturer’s instructions. Assessment of lineage-specific markers was carried out through both immunofluorescence (IF) and quantitative real time PCR (qRT-PCR) (Table S1).

### Quantitative real-time (qRT)-PCR

Total RNA was isolated using NZYol (NZYTech®, MB18501) and DNase I treatment (Roche^®^, 04716728001). cDNA was synthesised using the Transcriptor High Fidelity cDNA Synthesis Kit (Roche^®^, 5081963001) and amplified by qRT-PCR with specific primers for the targeted genes (Table S1), using the Universal SYBR Green Supermix (Bio-Rad, 1725274). All PCR reactions were performed in triplicate for 40 cycles using the ViiA™7 RT-PCR Systems (Applied BioSystems). The mRNA expression levels are depicted as the fold change of the target gene normalised against *GAPDH* (for *SeV* clearance) or *U6* (for differentiation markers) housekeeping genes (2^–ΔCt^).

### Immunofluorescence assays

Coverslip-seeded cells underwent fixation with 3.7% paraformaldehyde (PFA) in phosphate buffered saline (PBS), for 10 min at room temperature (RT) and subsequent permeabilization with 0.5% Triton X-100 in PBS, for 10 min at RT. Following this, cells were blocked with 5% FBS (Life Technologies, A5256701) in PBS, for 30 min at RT. Cells were then incubated overnight at 4 °C with the specified primary antibodies (Table S1); the following day, incubation with the corresponding secondary antibodies diluted in 1% FBS in PBS (Table S1), was performed for 1 h at RT and nuclei were then counterstained with DAPI (0.2 mg/ml; Cat# D9542, Sigma). Images were acquired using a Zeiss LSM 710 Confocal Laser Point-Scanning Microscope.

### Flow cytometry

iPSCs, dissociated into single cells, were fixed in 2% PFA, and stored at 4 °C. For intracellular staining, cells were permeabilized with 0.1% Saponin (Sigma, SAE0073), for 15 min at RT, and incubated with primary antibodies (OCT4 or SOX2) (Table S1) for 1 h at RT, after washing. 1% FBS/1xPBS was used to wash cells, twice, before further incubation for 45 min, in the dark, with the goat anti-Mouse IgG (H + L) Cross-Adsorbed Secondary Antibody, Alexa Fluor™ 488 (Thermo Fisher Scientific, A-11001). For surface staining, cells were resuspended in conjugated antibodies (SSEA4 or TRA1-60) (Table S1), diluted in 3% FBS/1xPBS, for 30 min at RT. Before analysis on the FACSCalibur™ flow cytometer (Becton Dickinson), all cells were resuspended in 1xPBS. In each experimental sample, at least 10,000 events were recorded within the defined gate based on side scatter (SSC) and forward scatter (FSC). Results were analysed using FlowJo software.

### Short tandem repeat (STR) analysis

To assess the clonality of each generated cell line, genomic DNA isolated from both iPSCs and the respective PBMCs, using phenol:chloroform:isoamyl alcohol extraction, was sent to Genomed SA (Lisbon, Portugal). There, STR DNA analysis was performed using the AmpFLSTR^®^ Identifiler^®^ Plus PCR Amplification Kit, which amplifies STR loci (D8S1179, D21S11, D7S820, CSF1PO, D3S1358, TH01, D13S317, D16S539, D2S1338, D19S433, vWA, TPOX, D18S51, D5S818, FGA), along with a gender-determining marker, Amelogenin (AMEL).

### Mycoplasma detection

iPSC cultures were checked for mycoplasma contamination with the qPCR Mycoplasma Test (Mycoplasmacheck, Eurofins Genomics), according to the manufacturer’s instructions.

## Results

### Clinical information

The patient is a 64-year-old female patient, diagnosed with familial HCM at the age of 15. She presented a maximal wall thickness of 21 mm (normal < 12 mm). Genetic testing identified her as heterozygous for the c.772G > A mutation in *MYBPC3*. This mutation co-segregated in the family, with 4 of 9 tested relatives sharing the same variant. Clinically, the patient presented biventricular and septal hypertrophy. Due to arrhythmia episodes, a cardioverter defibrillator was implanted as a preventive measure against sudden cardiac death. As the patient progressed to a burnout phase, leading to dilated cardiomyopathy and advanced heart failure, she underwent a heart transplant at the age of 63.

### Establishment and characterization of induced pluripotent stem cells carrying the MYBPC3: c.772G > A mutation

**Fig. 1:**
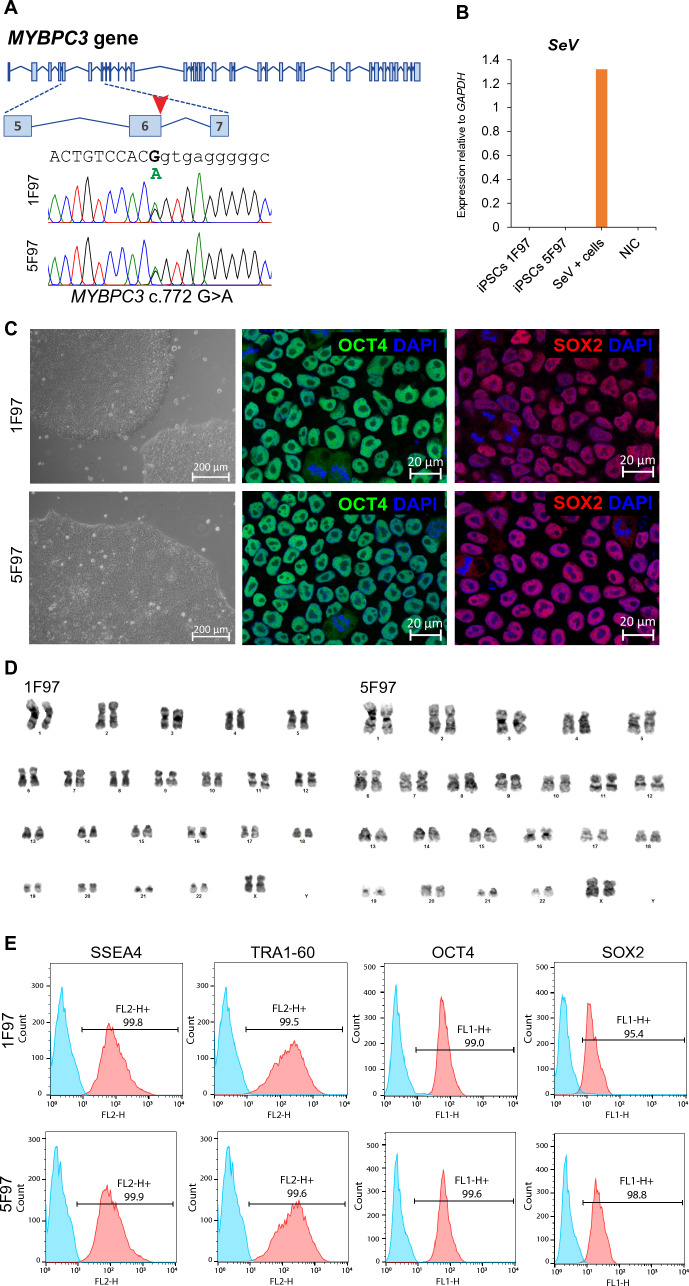
Characterization of the 1F97 (IBBISTi009-A) and 5F97(IBBISTi009-B) iPSC lines. **A** Schematic representation of *MYBPC3* c.772 region and Sanger sequencing results of generated iPSC lines. **B** qRT-PCR analysis of *SeV* expression in iPSCs clones, infected (Sev +) and non-infected cells (NIC). Relative expression was normalized against *GAPDH*. **C** On the left, a bright photomicrograph of iPSC colonies. Scale bar, 200 μm. On the right, immunofluorescence images of OCT4 and SOX2 staining. Scale bar, 20 μm. **D** Representative G-banding karyotype images of a normal 46, XX karyotype for both 1F97 (*n* = 30 metaphase spreads) and 5F97 (*n* = 13 metaphase spreads) iPSC lines. **E** Flow cytometry analysis of SSEA4, TRA-1-60, OCT4 and SOX2 pluripotent stem cell markers of generated iPSC lines

**Fig. 2:**
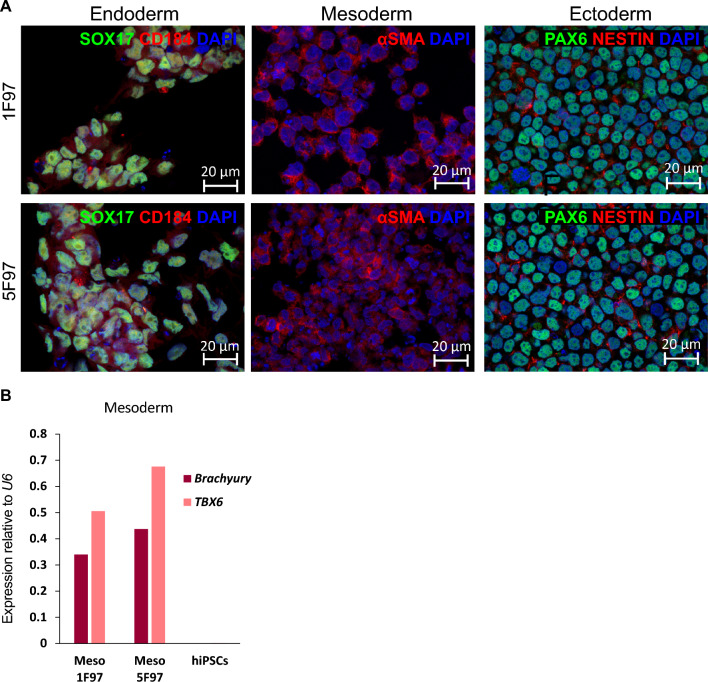
Trilineage differentiation potential of iPSC lines. **A** Representative immunofluorescence images of trilineage differentiation. Endoderm (SOX17 and CD184), Mesoderm (αSMA) and Ectoderm (PAX6 and NESTIN). Scale bar, 20 μm. **B** qRT-PCR analysis of expression of the mesoderm markers, *Brachyury* and *TBX6*, in both differentiated and non-differentiated cells (hiPSCs). Relative expression normalized to *U6*

PBMCs collected from the patient F97 were reprogrammed into iPSCs using a non-integrative method based on SeV transiently overexpressing the reprogramming factors OCT3/4, SOX2, KLF4, and c-MYC. Upon emergence of stem-like colonies, cells were transferred to Matrigel-coated plates and expanded in mTeSR™ Plus medium. Genomic DNA from two selected iPSC clones (1F97 and 5F97) was isolated, and Sanger sequencing confirmed the presence of the mutation in heterozygosity (Fig. 1A). *SeV* loss was confirmed by qRT-PCR, with *SeV* specific primers normalized to the housekeeping gene *GAPDH*, using PBMCs 3 days after SeV infection (SeV + cells) and noninfected cells (NIC) as positive and negative controls, respectively (Fig. 1B; Table S1). 1F97 and 5F97 exhibited stem-like morphology, positive immunofluorescence (IF) staining for OCT4 and SOX2 pluripotency markers (Fig. 1C) and a normal female 46, XX karyotype (Fig. 1D). Flow cytometry analysis shows that > 95% of cells express the pluripotency markers, SSEA4, TRA1-60, OCT4, and SOX2 (Fig. 1E). Differentiation potential of the newly generated iPSC lines was confirmed by direct differentiation into endoderm (IF staining for SOX17 and CD184) (Fig. 2A, Endoderm), mesoderm (IF staining for αSMA, positive for *Brachyury* and *TBX6* by qRT-PCR) (Fig. 2A, Mesoderm and Fig. 2B), and ectoderm (IF staining for PAX6 and NESTIN) (Fig. 2A, Ectoderm). Mycoplasma testing yielded negative results for both iPSC cultures (data not shown), and clonal identity was confirmed through STR DNA analysis of parental PBMCs and derived iPSCs (Table S2; original reports available with the authors).

## Discussion

We have successfully generated two novel iPSC lines from a Portuguese patient diagnosed with familial HCM at the age of 15, who was heterozygous for the *MYBPC3*: c.772G > A variant. The patient's clinical condition progressed to advanced heart failure, ultimately necessitating a heart transplant at the age of 63. A recent study investigated a cohort of patients from the Tuscany region, where this variant is highly prevalent due to a founder effect [18]. Among 93 HCM patients carrying the c.772G > A variant, the analysis revealed a mean age at diagnosis of 50 ± 15 years, with over 90% reporting either no symptoms or mild limitations [18]. Over a mean follow-up period of 6 ± 4 years, a trend towards disease progression after age 45 was observed, with over one-fifth of the patients showing advanced left ventricular dysfunction at final evaluation [18]. Given the atypical early onset and severity of the disease in the Portuguese patient highlighted in our study, the newly derived iPSC lines hold promise for uncovering the underlying factors contributing to this distinct presentation. It is worth noting that widespread clinical heterogeneity is a common feature of almost all genetic cardiovascular diseases, suggesting that factors beyond the primary mutation itself are important in modifying the clinical phenotype, either by exacerbating or protecting against the disease [23]. These modifying factors are poorly understood and may include environmental cues, age and gender-related influences, and secondary genetic variation [23]. Additionally, the novel iPSCs expand the existing repertoire of *MYPBC3*-mutated cell lines, broadening the spectrum of resources for exploring how diverse mutations induce HCM (Table 1). Previously reported iPSC lines harboring missense mutations include the c.655G > C (p.Val219Leu), the c.961G > A (p.Val321Met), and the c.1484G > A (p.Arg495Gln) variants [24, 25]. While haploinsufficiency is widely acknowledged as the predominant mechanism underlying disease in HCM-associated *MYBPC3* mutations, the precise manner in which these individual amino acid substitutions affect the functional properties of the mutant cMyBP-C protein within the sarcomeres is still unknown. Furthermore, whether any of these variants interferes with splicing, as shown for the c.772G > A mutation [19–22], remains to be investigated. Cardiomyocytes derived from patient-specific iPSCs (iPSC-CMs) with the c.655G > C, c.961G > A, and c.772G > A variants displayed consistent signs of impaired function, such as prolonged relaxation and action potentials, as well as aberrant Ca2 + handling [18, 24]. Nevertheless, the precise molecular pathways linking the primary sarcomeric mutation to the observed adaptive alterations in cardiomyocyte function remain largely elusive. The development of patient-specific iPSCs harboring distinct mutations presents a promising avenue for exploring these mechanisms in greater detail.

**Table 1:** iPSC lines derived from HCM patients

Gene	Mutation	Patient data	Comments	References
*MYH7*	Het c.1063G > A (p.Ala355Thr) missense	Chinese female	Mutation-corrected isogenic iPSCs; altered phenotype in iPSC-CMs	[26]
*MYH7*	Het c.1208G > A (p.Arg403Gln) missense	North American females	Telomere shortening in iPSC-CMs; allele-specific silencing in iPSC-CM	[27, 28]
*MYH7*	Het c.1324C > G (p.Arg442Gly) missense	North American female	Altered phenotype in iPSC-CMs	[29]
*MYH7*	Het c.1988G > A (p.Arg663His) missense	North American male and female; African American female	Altered phenotype in iPSC-CMs	[24, 27, 30]
*MYH7*	Het c.2093 T > C (p.Val698Ala) missense	Australian male	–	[31]
*MYH7*	Het c.2155C > T (p.Arg719Trp) missense	North American male	Altered phenotype in iPSC-CMs	[27]
*MYH7*	Het c.2167C > T (p.Arg723Cys) missense	North American male	Altered phenotype in iPSC-CMs	[27]
*MYH7*	Het c.727C > T (p.Arg243Cys) missense	Undisclosed	Increased myeloperoxidase levels in iPSC-CMs	[32]
*MYH7*	Het c.2543A > G (p.Glu848Gly) missense	North American male and female	Altered phenotype in iPSC-CMs	[33, 34]
*MYBPC3*	Het c.459del (p.Ile154fs) frameshift	Southeast Asian male	Causes HCM via chronic activation of nonsense-mediated decay	[35]
*MYBPC3*	Het c.655G > C (p.Val219Leu) missense	North American female	Altered phenotype in iPSC-CMs	[24]
*MYBPC3*	Het c.772G > A (p.Glu258Lys) missense	Italian male and North American individual(s)	Altered phenotype in iPSC-CMs	[18, 36]
*MYBPC3*	Het c.772 + 1G > A splice site	Chinese (Han) male	–	[37]
*MYBPC3*	Het c.961G > A (p.Val321Met) missense	North American female	Altered phenotype in iPSC-CMs	[24, 38]
*MYBPC3*	Het c.977G > A (p.Arg326Gln) missense	Russian male	Altered phenotype in iPSC-CMs	[39]
*MYBPC3*	Het c.1090 + 453C > T splice-disrupting intronic variant	Australian female	–	[40]
*MYBPC3*	Het c.1224-52G > A splice-disrupting intronic variant	Australian male	–	[40]
*MYBPC3*	Het c.1358-1359insC (p.Pro453fs) frameshift	German individual	Altered phenotype in iPSC-CMs	[41]
*MYBPC3*	Het c.1377del (p.Leu460fs) frameshift	Neonate Chinese female	–	[42]
*MYBPC3*	Het c.1484G > A (p.Arg495Gln) missense	Portuguese male and female	–	[25]
*MYBPC3*	Het c.1504C > T (p.Arg502Trp) missense	Chinese (Han) and North–west European males	–	[43, 44]
*MYBPC3*	Het c.1543-1545del (p.Asn515del) in frame deletion	Russian female	–	[45]
*MYBPC3*	Het c.1731G > A (p.Trp577Ter) nonsense	Portuguese male	–	[46]
*MYBPC3*	Het c.2373dupG (p.Trp792fs) frameshift	Dutch males	Altered phenotype in iPSC-CMs	[47]
*MYBPC3*	Het c.2670G > A (p.Trp890Ter) nonsense	Portuguese female	–	[46]
*MYBPC3*	Het c.2827C > T (p.Arg943Ter) nonsense	North American male and female	Isogenic lines; Altered phenotype in iPSC-CMs	[27, 48]
*MYBPC3*	Het c.2905 + 1G > A splice site	North American female	Altered phenotype in iPSC-CMs	[24]
*MYBPC3*	Het c.2997-3017del (p.Gly999-Gln1004) in frame deletion	Japanese female	Environmental factor endothelin-1 promoted the HCM pathological phenotype in iPSC-CMs	[49]
*MYBPC3*	Het c.3181C > T (p.Gln1061Ter) nonsense	Finnish males	Altered phenotype in iPSC-CMs	[50, 51]
*MYBPC3*	Het c.3217dupC (p.Arg1073fs) frameshift	North American individual	Isogenic lines; Altered phenotype in iPSC-CMs	[48]
*MYBPC3*	Het c.3330 + 2 T > G splice site	North American male and female (not Hispanic or Latino)	–	[52]
*MYBPC3*	Het c.3369-3370insC (p.Cys1124fs) frameshift	Chinese (Han) male	–	[53]
*MYBPC3*	Het c.3764C > A (p.Ala1225Asp) missense	Chinese (Han) male	–	[54]
*MYBPC3*	Het *MYBPC3*Δ25bp in frame deletion	South Asian descendants	Altered phenotype in iPSC-CMs in association with novel *MYBPC3* variant, D389V	[55]
*ACTC1*	Het c.301G > A (p.Glu99Lys) missense	UK males	Isogenic lines; Altered phenotype in iPSC-CMs	[56]
*ACTN2*	Het c.740C > T (p.Thr247Met) missense	German family	Isogenic lines; Altered phenotype in iPSC-CMs	[57]
*ALPK3*	Het c.2023delC (p.Gln675fs) frameshift	East Asian males	–	[58]
*JPH2*	Het c.482C > A (p.Thr161Lys) missense	Finnish male	Isogenic lines; Altered phenotype in iPSC-CMs	[59]
*MYL2*	Het c.173G > T (p.Arg58Leu) missense	North American female	Altered phenotype in iPSC-CMs	[60]
*MYL3*	Het c.170C > G (p.Ala57Asp) missense	North American individual	Altered phenotype in iPSC-CMs	[38]
*TNNT2*	Het c.274 C > T (p.Arg92Trp) missense	North American female	Altered phenotype in iPSC-CMs	[24]
*TPM1*	Het c.523G > A (p.Asp175Asn) missense	Finnish male and female	Altered phenotype in iPSC-CMs	[50, 51]

*MYH7* β-myosin heavy chain gene, *MYBPC3* Cardiac myosin-binding protein C gene, *ACTC1* Cardiac α-actin gene, *ACTN2* actinin alpha 2 gene, *ALPK3* Alpha Kinase 3 gene, *JPH2* Junctophilin 2 gene, *MYL2* Myosin light chain 2 gene, *MYL3* Myosin light chain 3 gene, *TNNT2* Cardiac troponin T gene, *TPM1* α-tropomyosin gene, *Het* Heterozygoty, *Ala* Alanine, *Arg* Arginine, *Asn* Asparagine, *Asp* Aspartic acid, *Cys* Cysteine, *Gln* Glutamine, *Gly* Glycine, *Glu* Glutamic acid, *His* Histidine, *Ile* Isoleucine, *Leu* Leucine, *Lys* Lysine, *Met* Methionine, *Pro* Proline, *Thr* Threonine, *Val* Valine, *Trp* Tryptophan, *c* coding, *p* protein

### Supplementary Information

Below is the link to the electronic supplementary material.

Supplementary file1 (DOCX 82 KB)

## Data Availability

The data that support the findings of this study are available on request from the corresponding author S.M.
