# Supplementary material for: Generation of induced pluripotent stem cells from an individual with early onset and severe hypertrophic cardiomyopathy linked to MYBPC3: c.772G > A mutation
Source: Hum Cell. 2024 May 18;37(4):1205–14. doi: 10.1007/s13577-024-01073-y (PMC11194200; doi:10.1007/s13577-024-01073-y)
Supplement: Supplementary file 1 — Supplementary file1 (DOCX 82 KB) [file 13577_2024_1073_MOESM1_ESM.docx]

**Table S1**- Reagents details (primers and antibodies used)

|  | **Antibodies used for immunocytochemistry/flow-cytometry** | | | |
| --- | --- | --- | --- | --- |
|  | **Antibody** | **Dilution** | **Company Cat #** | **RRID** |
| Pluripotency Markers (FC) | Mouse anti-OCT4 | 1:400 | Millipore, MAB4419 | AB_1977399 |
|  | Mouse anti-SOX2 | 1:200 | R&D Systems, MAB2018 | AB_358009 |
|  | Mouse anti-SSEA4 (PE) | 1:20 | Miltenyi Biotec, 130–098–369 | AB_2653519 |
|  | Mouse anti-TRA1-60 (PE) | 1:10 | Miltenyi Biotec, 130–100–347 | AB_2654227 |
| Pluripotency Markers (IF) | Mouse anti-OCT4 | 1:200 | Millipore, MAB4419 | AB_1977399 |
|  | Mouse anti-SOX2 | 1:200 | R&D Systems, MAB2018 | AB_358009 |
| Differentiation Markers (IF) | Rabbit anti-PAX6 | 1:400 | Biolegend, 901301 | AB_2565003 |
|  | Mouse anti-NESTIN | 1:400 | R&D Systems, MAB2736 | AB_2282664 |
|  | Rabbit anti-SOX17 | 1:200 | Abcam, ab224637 | AB_2801385 |
|  | PE Mouse anti-CD184 | 1:20 | Biolegend, 306506 | AB_314612 |
|  | Mouse anti-αSMA | 1:100 | SIGMA, A2547 | AB_476701 |
| Secondary antibodies (FC) | Goat anti-mouse IgG, Alexa  Fluor 488 | 1:1000 | Thermo Fisher Scientific, A- 11001 | AB_2534069 |
| Secondary antibodies (IF) | Goat anti-mouse IgG, Alexa  Fluor 546 | 1:500 | Thermo Fisher Scientific A- 11003 | AB_2534071 |
|  | Goat anti-mouse IgG, Alexa  Fluor 488 | 1:500 | Thermo Fisher Scientific, A- 11001 | AB_2534069 |
|  | Goat anti-rabbit IgG, Alexa  Fluor 488 | 1:500 | Thermo Fisher Scientific A-11034 | AB_2576217 |
|  | **Primers** | | | |
|  | **Target** | **Size of band** | **Forward/Reverse primer (5′-3′)** | |
| Sendai reprogramming vector (qRT-PCR) | *SeV* |  | GGATCACTAGGTGATATCGAGC/ ACCAGACAAGAGTTTAAGAGATATGTATC | |
| House-Keeping Genes (qRT-PCR) | *GAPDH* |  | TCGTGGAGTCCACTGGCGTC/ TCATGAGTCCTTCCACGATAC | |
|  | *U6* |  | CGTTCGGCAGCACATATAC/  AAATATGGAACGCTTCACGA | |
| Differentiation Markers (qRT-PCR) | *Brachyury* |  | TCAGCAAAGTCAAGCTCACCA/  CCCCAACTCTCACTATGTGGATT | |
|  | *TBX6* |  | ACCGTGTCTACATTCACCCC/  CACGATGGAAAGACACAGGC | |
| Targeted mutation analysis (PCR) | *MYBPC3* c.772 G>A |  | GCCACTCCCAGTCTCCTTTA/ GGCATCCTCCTTAGTGTTGG | |

**Table S2** –STR DNA analysis of PBMCs isolated from the blood of F97 patient and of the two generated iPSC lines (1F97 and 5F97). Identified alleles of each amplified STR loci are discriminated. Along with a gender-determining marker, Amelogenin (AMEL).
